# Isoform-Specific Effects of Apolipoprotein E on Markers of Inflammation and Toxicity in Brain Glia and Neuronal Cells In Vitro

**DOI:** 10.3390/cimb43010018

**Published:** 2021-05-27

**Authors:** Jaclyn Iannucci, Abhik Sen, Paula Grammas

**Affiliations:** 1 George & Anne Ryan Institute for Neuroscience, University of Rhode Island, Kingston, RI 02881, USA; abhiksen78@gmail.com (A.S.); pgrammas@uri.edu (P.G.); 2 Department of Biomedical and Pharmaceutical Sciences, College of Pharmacy, University of Rhode Island, Kingston, RI 02881, USA; 3 Department of Molecular Biology, ICMR–Rajendra Memorial Research Institute of Medical Sciences, Indian Council of Medical Research, Agam Kuan, Patna 800007, India

**Keywords:** apolipoprotein E, neuroinflammation, toxicity, Alzheimer’s disease

## Abstract

Mutations to the cholesterol transport protein apolipoprotein E (ApoE) have been identified as a major risk factor for the development of sporadic or late-onset Alzheimer’s disease (AD), with the *e4* allele representing an increased risk and the rare *e2* allele having a reduced risk compared to the primary *e3* form. The reasons behind the change in risk are not entirely understood, though ApoE4 has been connected to inflammation and toxicity in both the brain and the periphery. The goal of this study was to better understand how the ApoE isoforms (ApoE2/3/4) confer differential AD-related risk by assessing cell-specific ApoE-related neuroinflammatory and neurotoxic effects. We compared the effects of ApoE isoforms in vitro on human astrocytes, a human immortalized microglia cell line (HMC3), and the human neuroblastoma cell line SH-SY5Y. Cells were treated for 24 h with or without recombinant ApoE2, ApoE3, or ApoE4 (20 nM) and inflammation and toxicity markers assessed. Our results indicated the expression of inflammatory cytokines IL-1β, TNFα, and IL-6 in human astrocytes was increased in response to all ApoE isoforms, with ApoE4 evoking the highest level of cytokine expression. In response to ApoE2 or ApoE3, microglial cells showed reduced levels of microglial activation markers TREM2 and Clec7a, while ApoE4 induced increased levels of both markers. ApoE2 promoted neuron survival through increased BDNF release from astrocytes. In addition, ApoE2 promoted, while ApoE4 reduced, neuronal viability. Overall, these results suggest that ApoE4 acts on cells in the brain to promote inflammation and neuronal injury and that the deleterious effects of ApoE4 on these cells may, in part, contribute to its role as a risk factor for AD.

## 1. Introduction

The most significant known genetic risk factor for sporadic or late-onset Alzheimer’s disease (AD) is an allele variant of apolipoprotein E (ApoE), a protein involved in lipid metabolism and cholesterol homeostasis [1]. The ApoE gene has three polymorphic alleles that occur in humans, *e2*, *e3*, and *e4.* These alleles occur at different frequencies, with *e3* occurring most frequently at 65–70%, followed by *e4* and *e2* [1]. However, the frequency of the *e4* allele is substantially increased by approximately 40% in AD patients compared to healthy counterparts [2]. Population studies have shown that the *e4* allele is associated with a significantly elevated likelihood for developing Alzheimer’s, while the *e2* variant seems to have protective effects compared to *e3* [1,2]. Although the relationship between ApoE alleles and AD has been well-established, the mechanisms behind these risk differences have not been well characterized.

ApoE is primarily synthesized in the liver, but the brain synthesizes its own ApoE as well. ApoE mRNA has been found in the cerebral cortex, hippocampus, cerebellum, and medulla [3]. In the brain, ApoE is synthesized primarily by astrocytes, but is also believed to be produced by oligodendrocytes, microglia, and neurons under stress conditions [1]. Due to its primary function as a lipid transport molecule, ApoE has largely been studied for its role in Aβ clearance in AD. ApoE4 impairs Aβ clearance compared to ApoE3, and increases amyloid formation in the brain [4]. Brain plaque load is the lowest in ApoE2 and the highest in ApoE4 individuals [2]. More recent research, however, is focusing on diverse roles for ApoE in the brain as a mediator of cerebrovascular function, neuroinflammation, synapse formation, and neurotoxicity [2].

A clear role for ApoE in the modulation of the innate immune system has been identified. Neuroinflammation is known to play a key role in the pathological progression of AD [5,6,7], and ApoE4 is associated with pro-inflammatory changes in both the central nervous system (CNS) and periphery [8]. Post-mortem ApoE4-expressing brain samples exhibit upregulation in proteins related to the regulation of inflammatory response, compared to both ApoE3 and ApoE2-expressing tissue [9]. Specifically, a clear role for mediating microglia phenotype and function has been identified for ApoE. Microglia expressing human ApoE4 exhibit significantly increased production of pro-inflammatory mediators, including tumor necrosis factor-α (TNFα), interleukin (IL)-6, and nitric oxide (NO) [10,11,12,13]. More recent research using iPSC-derived microglia expressing human ApoE found that ApoE4 microglia have impaired migration and reduced phagocytic function [14]. Further support for the ability of ApoE to regulate neuroinflammatory processes comes from the finding that ApoE signaling via triggering receptor expressed on myeloid cells 2 (TREM2) regulates a switch in microglia towards a neurodegenerative phenotype. Targeting ApoE signaling has the ability to restore homeostatic function in microglia [15]. Therefore, ApoE modulation of the innate immune system, particularly through alterations in microglia phenotype, may play a key role in ApoE-related AD pathology.

ApoE isoforms may also regulate neuronal survival or toxicity, a key aspect of neurodegenerative pathological processes in AD. Directly, treatment of iPSC-derived neurons with recombinant ApoE2/3/4 stimulates various signal transduction cascades with different levels of potency. One such cascade is APP synthesis and synapse formation, which is enhanced in ApoE4 treated neurons and reduced in ApoE2 treated compared to ApoE3 [16]. ApoE4 has been shown to promote tau phosphorylation and neurotoxicity [2], as well as impair synaptogenesis [3]. ApoE4 is more susceptible than other isoforms to proteolytic cleavage, and this cleavage fragment is neurotoxic [17]. Indirectly, ApoE may influence synapse formation and neuron survival through astrocyte activity. ApoE3 enhances astrocyte phagocytosis of synapses, while ApoE4 decreases the same activity [18]. Astrocytes are also a primary producer of neurotrophic support, including brain derived neurotrophic factor (BDNF). BDNF is decreased in the serum and brain of AD patients compared to healthy controls [19,20], and ApoE isoforms have been found to modulate astrocyte production and secretion of BDNF [21]. Overall, these findings further indicate that ApoE isoform-specific effects on cells in the brain, including neurons and astrocytes, may be responsible for the increased ApoE4-related AD risk.

The goal of this study was to better understand how the ApoE isoforms (ApoE2/3/4) confer differential AD-related risk by assessing cell-specific ApoE-related neuroinflammatory and neurotoxic effects. Human microglia, astrocytes, and neurons were treated with ApoE2/3/4 in vitro, and the effects of the ApoE isoforms on markers related to neuroinflammation and neurotoxicity were assessed. Our findings help to better characterize the isoform and cell-specific effects of ApoE that may contribute to the development of AD-related inflammation and neuron death.

## 2. Materials and Methods

### 2.1. Cells and Other Reagents

Human immortalized microglia cell line (HMC3; CRL-3304) and Eagle’s Minimum Essential Medium (EMEM; 30-2003) were purchased from ATCC (Manassas, VA, USA). Human primary astrocytes (#1800) and astrocyte medium (#1801) along with astrocyte growth supplements were purchased from ScienCell Research Laboratories (Carlsbad, CA, USA). Human neuroblastoma cells (SH-SY5Y) were kindly provided by Dr. Navindra Seeram (University of Rhode Island, Kingston, RI, USA). Dulbecco’s Modified Eagle medium (DMEM), DMEM/F12, fetal bovine serum (FBS), bovine serum albumin (BSA), poly-L-lysine, and recombinant human ApoE2 (rh-ApoE2), ApoE3 (rh-ApoE3), and ApoE4 (rh-ApoE4) were purchased from Sigma Aldrich (St. Louis, MO, USA).

### 2.2. Cell Culture and Treatments

HMC3 were grown and maintained in EMEM with 10% FBS. Treatment media was serum free EMEM. Human primary astrocytes were plated on poly-L-lysine coated plates and maintained in astrocyte medium with growth supplements and 2%FBS, according to protocols provided by ScienCell. For treatments, media was replaced with serum-free DMEM. SH-SY5Y were grown and maintained in DMEM/F12 with 10% FBS, and treatments were done in serum-free DMEM/F12.

Rh-ApoE2/3/4 were dissolved in PBS pH 7.4 to make a stock at 6 μM. All cells were treated with cholesterol (100 µM) (control) with or without 20 nM rh-ApoE2/3/4 for 24 h. ApoE and cholesterol were added separately to cultures. The concentration of ApoE was selected based on previous results with both human astrocytes and SH-SY5Y [21,22]. Cholesterol was utilized for these experiments because evidence suggests the effects of ApoE isoforms are determined by their lipidation state [23].

### 2.3. Cell Viability and Toxicity

The viability of SH-SY5Y was assessed following treatments with rh-ApoE2/3/4. Cells were seeded in 96-well plates at 100,000 cells/mL, and were allowed to adhere for 48 h. Cells were treated with cholesterol with or without rh-ApoE2/3/4 for 24 h. Cellular viability was determined as a percentage of control (cholesterol) by CellTiter 96 **^®^** Aqueous One Solution Cell Proliferation Assay (MTS; Promega, Madison, WI, USA), and read for absorbance at 490 nm on the Synergy HTX multi-mode reader (Biotex Instruments, Winooski, VT, USA).

Cellular toxicity of SH-SY5Y following treatments was assessed by measuring lactate dehydrogenase (LDH) release in the conditioned media. Supernatant from cells was collected and total LDH was assessed using a cytotoxicity detection kit (Millipore Sigma, Burlington, MA, USA). Samples were incubated with chromogenic dye and catalyst, then absorbance was read at 490 nm using a Synergy HTX multi-mode reader (Biotek Instruments, Winooski, VT, USA).

### 2.4. Western Blot

Western blot was used to measure protein expression in astrocytes and microglia following treatment with rh-ApoE2/3/4. Cellular protein was extracted as previously described [24]. Briefly, cells were lysed in Tris-HCl buffer with protease inhibitors. Lysates were mixed with Sample Buffer (BioRad, Hercules, CA, USA) and samples were run by SDS-PAGE on gradient gels (Invitrogen, Carlsbad, CA, USA). For all Western blots, 25 μg total protein was loaded for each well. Samples were transferred to nitrocellulose membranes (iBlot, Invitrogen, Carlsbad, CA, USA). All membranes were blocked and incubated with primary antibody overnight at 4 °C. For BDNF Western blots, secreted media was collected from treated astrocytes and concentrated 10-fold as previously described [21]. Samples were run using the same method as outlined above, with 40 μg of total protein loaded for each well. Primary antibodies include IL-6 (Bioss, Woburn, MA, USA; bs-4539R; 1:500), TNFα (Novus Biologicals, Littleton, CO, USA; NBP1-19532; 1:500), IL-1β (Bioss, Woburn, MA, USA; bs-6319R; 1:1000), TREM2 (Novus Biologicals, Littleton CO, USA; NBP1-07101; 1:500), Clec7a (Novus Biologicals, Littleton, CO, USA; NB01-45514; 1:500), BDNF (Abcam, Cambridge, MA, USA; ab108319; 1:1000), and β-Actin (Santa Cruz, Dallas, TX, USA; AC-15; 1:10,000). Bound antibody was detected by infrared secondary antibodies (Li-COR, Lincoln, NE, USA; 1:10,000), and imaged on LiCor Odyssey (Li-COR Biosciences, Lincoln, NE, USA). Quantification of Western blots was done in ImageJ, and values for each protein were normalized to β-Actin on the same blot.

### 2.5. Statistical Analysis

All data were analyzed for significance using one-way analysis of variance (ANOVA) and multiple comparisons post-hoc were completed using Bonferroni test on GraphPad Prism (version 8.04; GraphPad, San Diego, CA, USA). Data are represented as Mean ± SEM, groups contain N = 3 unless otherwise specified. Statistical significance was set at *p* ≤ 0.05 for all experiments.

## 3. Results

### 3.1. ApoE4 Increases Inflammatory Cytokine Expression in Human Astrocytes

ApoE4 is associated with increased inflammation in various model systems and disease states [8,25,26]. Recently, the regulation of cholesterol by ApoE4 in the brain has been shown to be cell-type specific [27]. While astrocytes have been widely studied for their role in ApoE4-related lipid homeostasis and glucose metabolism [28], less is known about their function in ApoE4-associated neuroinflammation. Here we explored the ability of ApoE isoforms to affect the production of pro-inflammatory cytokines in human astrocytes.

**Figure 1:**
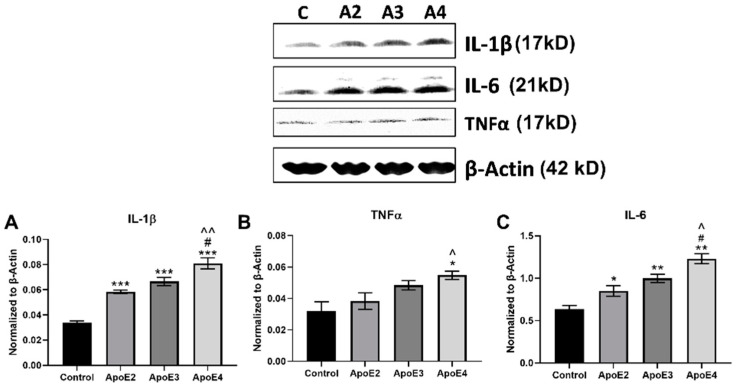
Expression of inflammatory cytokines by astrocytes treated with ApoE isoforms. Human primary astrocytes were grown in vitro and treated for 24 h with 100 µM cholesterol (control) with or without recombinant ApoE2/3/4 (20 nM). Following treatment, cell lysate was collected and Western blot was used to assess the expression of inflammation-related proteins: (**A**) IL-1β, (**B**) TNFα, and (**C**) IL-6. N = 3, * *p* < 0.05 vs. Control, ** *p* < 0.01 vs. Control, *** *p* < 0.001 vs. Control, ^#^ *p* < 0.05 vs. ApoE3, ^ *p* < 0.05 vs. ApoE2, ^^ *p* < 0.01 vs. ApoE2.

Intracellular expression of inflammatory cytokines IL-1β (Figure 1A), TNFα (Figure 1B), and IL-6 (Figure 1C) in human astrocytes following treatment with rh-ApoE2/3/4 was evaluated by Western blot. Treatment of astrocytes with all three ApoE isoforms significantly (*p* < 0.001) increased IL-1β expression compared to control cells (Figure 1A). However, ApoE4 evoked the highest level of IL-1β, which was significantly higher than that observed with ApoE 2 (*p* < 0.01). ApoE2 and ApoE3 both appeared to elicit a small but not significant increase in TNFα. In contrast, ApoE4 significantly (*p* < 0.05) increased TNFα compared to control, as well as to ApoE2 (Figure 1B). The ability of ApoE isoforms to affect IL-6 expression followed a pattern similar to that observed for IL-1β. Treatment of astrocytes with all three ApoE isoforms significantly (*p* < 0.01) increased IL-6 expression compared to untreated control cells (Figure 1C). However, ApoE4 evoked the highest level of IL-6, which was significantly (*p* < 0.05) higher than that observed with either ApoE2 or ApoE3.

### 3.2. ApoE4 Enhances the Inflammatory Activation of Microglia

**Figure 2:**
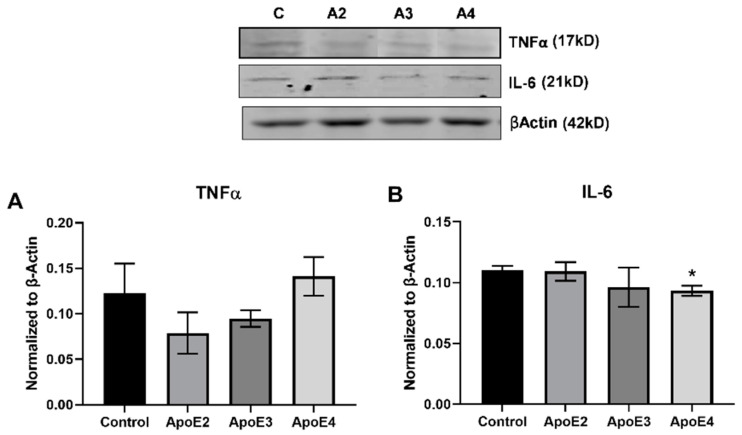
Expression of inflammatory cytokines by microglia treated with ApoE isoforms. Human immortalized microglia cells (HMC3) were grown in vitro and treated for 24 h with 100 µM cholesterol (control) with or without recombinant ApoE2/3/4 (20 nM). Following treatment, cell lysate was collected and Western blot was used to assess the expression of pro-inflammatory cytokines: (**A**) TNFα and (**B**) IL-6. N = 3, * *p* < 0.05 vs. Control.

**Figure 3:**
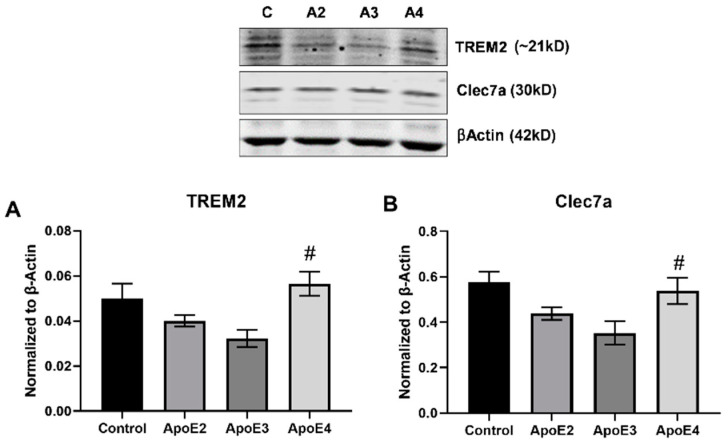
Expression of microglia activation markers following treatment with ApoE isoforms. Human immortalized microglia cells (HMC3) were grown in vitro and treated for 24 h with 100 µM cholesterol (control) with or without recombinant ApoE2/3/4 (20 nM). Following treatment, cell lysate was collected and Western blot was used to assess the expression of markers related to disease-associated microglia: (**A**) TREM2 and (**B**) Clec7a. N = 3, ^#^ *p* < 0.05 vs. ApoE3.

Microglia exhibit a range of phenotypes and activation states in response to extracellular signals. We examined the effect of treatment with ApoE isoforms on the human microglial cell line HMC3. Western blot was used to measure the expression of cytokines and markers related to inflammation (Figure 2) and microglial activation (Figure 3).

We examined the effect of ApoE isoforms on the production of both the pro-inflammatory cytokine TNFα as well as on IL-6, a complex cytokine with both pro- and anti-inflammatory properties. Treatment with ApoE4 increases TNFα level (ApoE2 vs. ApoE4 *p* = 0.0915; ApoE3 vs. ApoE4 *p* = 0.1911), while exposure to ApoE2 appeared to decrease TNFα expression compared to control (Figure 2A). In contrast, neither ApoE2 nor ApoE3 affected IL-6 expression while ApoE4 significantly (*p* < 0.05) decreased IL-6 expression compared to control (Figure 2B).

Microglia have been shown to take on an alternate activation state in AD, termed disease-associated microglia (DAM) [15,29]. DAMs are characterized by decreased expression of homeostatic markers and increased expression of a sub-set of inflammatory indicators. HMC3 were treated as described above and Western blot was used to assess the expression of markers related to microglia activation, TREM2 and Clec7a (Figure 3A,B). The data showed that treatment with either ApoE2 or ApoE3 diminished expression of TREM2 and Clec7a compared to control, while ApoE4 exposure increased levels of both TREM2 (Figure 3A) and Clec7a (Figure 2B). Expression of both TREM2 and Clec7a was significantly (*p* < 0.05) elevated in the ApoE4 treated group compared to ApoE3. Expression levels for TREM2 and Clec7a were also increased compared to ApoE2, though the difference was not significant (*p* = 0.1043 and *p* = 0.257, respectively). Both TREM2 and Clec7a expression levels were unchanged compared to control (Figure 3A,B).

### 3.3. ApoE2 Promotes Neuron Survival While ApoE4 Reduces Neuronal Viability

**Figure 4:**
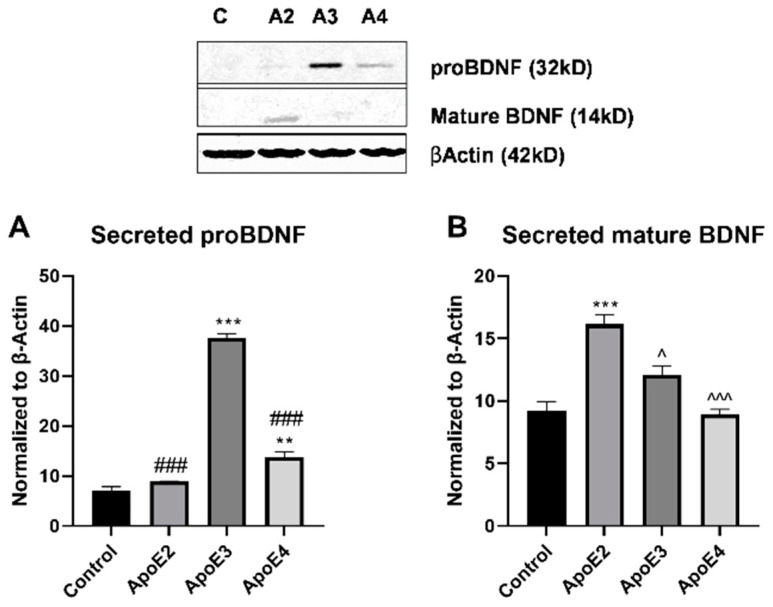
Evaluation of neurotrophic factors following treatment with ApoE isoforms. Human primary astrocytes were grown in vitro and treated for 24 h with 100 µM cholesterol (control) with or without recombinant ApoE2/3/4 (20 nM). Following treatment, astrocyte supernatant was collected, concentrated 10-fold, and Western blot was used to assess the expression of secreted BDNF. Expression was evaluated for both the (**A**) proBDNF and (**B**) mature BDNF isoforms. N = 3, ** *p* < 0.01 vs. Control, *** *p* < 0.001 vs. Control, ^###^ *p* < 0.001 vs. ApoE3, ^ *p* < 0.05 vs. ApoE2, ^^^ *p* < 0.001 vs. ApoE2.

Studies have indicated reduced levels of the neurotrophic factor BDNF in AD patients compared to healthy controls [19,20]. Because astrocytes are a primary source of BDNF in the brain, we investigated the connection between ApoE isoform and BDNF expression levels. Human astrocytes were treated with rh/ApoE2/3/4 for 24 h, conditioned-media collected, and BDNF expression level evaluated by Western blot (Figure 4). Expression of proBDNF was significantly (*p* < 0.001) elevated in ApoE3 treated cells compared to control, ApoE2, and ApoE4 groups. ProBDNF expression was also significantly (*p* < 0.01) elevated in ApoE4 treated cells compared to control. ApoE2 treatment did not alter the secretion of proBDNF compared to control (Figure 4A). Mature BDNF was virtually undetectable in the conditioned-media collected from control astrocytes. Treatment of astrocytes with ApoE2 significantly (*p* < 0.001) increased the level of mature BDNF detected in the secreted media (Figure 4). ApoE3 appeared to slightly increase mature BDNF secretion, although to a significantly (*p* < 0.05) lower extent than ApoE2. Exposure of astrocytes to ApoE4 had no effect on release of mature BDNF, with levels again undetectable (Figure 4B).

To further investigate the potential neuroprotective and neurotoxic effects of ApoE isoforms, we directly exposed the neuronal cell line SH-SY5Y to rh-ApoE2/3/4 for 24 h and assessed cellular viability (Table 1). Cellular viability was significantly (*p* < 0.01) increased in ApoE2 treated SH-SY5Y compared to control, with an increase of 14.9% ± 0.663. Viability was significantly (*p* < 0.001) decreased in ApoE4 compared to both ApoE2 and ApoE3, with a decrease of 9.7% ± 4.202 compared to control (Table 1). SH-SY5Y cells exposed to ApoE isoforms were also analyzed for cellular toxicity. Neither ApoE2 nor ApoE3 had any effect on release of lactate dehydrogenase (LDH). In contrast, ApoE4 treated cells display significantly (*p* < 0.05) increased cytotoxicity compared to untreated controls, with an increase in LDH release of 14% ± 4.164 (Table 1). Together, these findings indicate that ApoE2 is neuroprotective through both increased BDNF and direct action on neurons, while ApoE4 is likely neurotoxic through both direct and indirect mechanisms. 

## 4. Discussion

ApoE4 is the strongest known genetic risk factor for late-onset AD, while ApoE2 appears to be protective. While the different levels of risk related to each isoform have been well-established, the mechanism responsible for this relationship is still not entirely understood. Here, we investigated the cell type-specific effects of ApoE2/3/4 in the brain on markers related to neuroinflammation and neurotoxicity. Our results showed that ApoE acts in an isoform-specific manner to modulate pro-inflammatory activity of microglia and astrocytes, and affects neuron survival through both direct and indirect mechanisms.

We investigated the expression of pro-inflammatory cytokines by both microglia and astrocytes following treatment with ApoE isoforms. ApoE4 treatment increased the production of TNFα in relation to both ApoE2 and ApoE3, but significantly reduced the expression of IL-6 in microglia. While the pro-inflammatory activation of microglia by ApoE4 had been well-established, less is known about the ApoE-related inflammatory effects in astrocytes. Astrocytes, like microglia, are key regulators of the brain’s inflammatory response, and have been implicated in AD-related inflammatory and pathological processes [30]. Astrocytes have been found to produce inflammatory cytokines, including IL-6 and TNFα, following traumatic injury and ischemia [31]. Here, we show that ApoE treatment significantly increases the production of pro-inflammatory cytokines IL-1β, IL-6, and TNFα in a dose-dependent manner, with ApoE2 representing the lowest cytokine expression and ApoE4 representing the highest. TNFα is a key mediator in neuropathology and has been found to promote excitotoxicity, synaptic loss, and exacerbated amyloidogenesis in AD. Additionally, there is evidence to suggest elevated serum levels of TNFα in AD [32,33]. The production of TNFα is increased in both activated microglia and astrocytes in response to injury [32], and here TNFα expression is increased by ApoE4 in both cell types. Similar to TNFα, IL-1β is a pleiotropic cytokine that amplifies immune reactions [34] and is elevated in the CSF of AD patients [35]. Both TNFα and IL-1 can induce increased production of IL-6 and other inflammatory mediators via NF-κβ activation [33]. Mice with astrocyte-specific enhanced IL-6 production exhibit neurodegeneration and demyelination, along with decreased hippocampal long-term potentiation and learning impairments. Microglia in these mice also have enhanced pro-inflammatory activation [36]. However, IL-6 signaling is largely context dependent, and has been shown to mediate both pro-inflammatory and anti-inflammatory, protective processes [37], which may in part help to explain the reduction in IL-6 production by ApoE4-treated microglia. These findings highlight a previously underexplored role for ApoE in modulating the inflammatory state of astrocytes, and suggest that ApoE4 may influence AD pathology via increased production of inflammatory cytokines in both astrocytes and microglia.

These data also suggest ApoE-related differential activation of microglia. HMC3 microglia were treated with rh-ApoE2/3/4 and markers related microglial activation state were assessed. DAM refers to a subset of microglia identified in the AD brain that have dysregulated expression of sensing, house-keeping, and host-defense genes [15,29,38]. DAM are largely localized around Aβ plaques and display elevation of lipid metabolism and phagocytic activity-related genes, as well as genes related to increased AD risk [15]. ApoE is known to play a key role in the phenotypic of switch of microglia through signaling at TREM2 [15]. Here, we further the idea that ApoE4 enhances activation of the DAM phenotype by showing that ApoE4 treatment increased the expression of both TREM2 and the downstream, DAM-related protein Clec7a. This suggests that ApoE4 may confer increased risk for AD through increased activation of disease-associated microglia, and related, down-stream pro-inflammatory and disease progressing effects. It is interesting to note that expression of both TREM2 and Clec7a were not significantly different in ApoE4 treated cells when compared to control, suggesting that ApoE2 or ApoE3 may be acting in a protective manner to lower the baseline expression of these proteins.

Finally, our findings support the idea that ApoE2 is neuroprotective while ApoE4 is neurotoxic through both direct and indirect mechanisms. Human neuroblastoma cells (SH-SY5Y) were treated with rh-ApoE2/3/4 and cellular viability was assessed. ApoE2 significantly increased, while ApoE4 significantly decreased, viability. Synapse loss and progressive neuron death are core pathological hallmarks of AD, and these losses correlate with cognitive impairment [39]. Therefore, ApoE4 may help to promote AD pathological progression by enhancing neuron death, while ApoE2 protects against these same processes. Additionally, human astrocytes were treated with rh-ApoE2/3/4 and the secretion of the neurotrophic factor BDNF and its pro-form was assessed. Secretion of growth factors, including BDNF, is a major function of astrocytes [30]. BDNF has been found to be reduced in human AD, and a previous study found that ApoE isoforms modulated astrocyte production of specific BDNF isoforms [19,20,21]. Here, we show that ApoE2 significantly increased astrocyte secretion of mature BDNF. Peripheral BDNF has been found to protect against AD, with one standard deviation higher serum BDNF conferring a 33% decreased risk for AD [40]. BDNF enhances neurogenesis and neurotransmission, promotes synaptic growth and plasticity, and increases hippocampal long-term potentiation and related memory formation [19]. Additionally, BDNF has been found to have anti-apoptotic and anti-oxidant functions in experimental models of neurodegenerative disease [41]. On the other hand, both ApoE3 and ApoE4 exhibited an increased secretion of the proBDNF isoform. We have previously reported similar findings, suggesting that ApoE2 may enhance the cleavage of proBDNF to the mature form [21]. Unlike mature BDNF, proBDNF has been linked with the promotion of long-term depression and cell death [42]. Taken together, these findings support the hypothesis that ApoE2 reduces risk for AD by acting in a neuroprotective manner, through both direct and indirect action at neurons. On the other hand, ApoE4 may further promote AD-related risk through its neurotoxic effects in the brain.

While our findings support a pro-inflammatory and neurotoxic role for ApoE4, along with a neuroprotective one for ApoE2, it is important to interpret these results with a degree of caution. These data represent a relatively small sample size (*n* = 3) in a simplified in vitro system. While this system is advantageous in that it allows for the assessment of cell-specific ApoE-related effects, it is likely that ApoE-related mechanisms are more complicated in a complex in vivo system in which these (and other) cell types can communicate with each other. Newer models may help to better model this intracellular communication in vitro [43]. Additionally, immortalized cell lines were utilized for both microglia and neurons, and these responses may not perfectly mirror that of primary cells. Therefore, these findings should be interpreted as preliminary, and further investigation should be conducted to more clearly characterize the cell- and isoform-specific effects of ApoE in the brain. Such further studies may also help to further characterize the therapeutic potential of targeting ApoE signaling at various cell types. Recent studies have highlighted the therapeutic potential of targeting ApoE4, with one such study finding that ApoE immunotherapy was able to ameliorate amyloid-related pathology and protect cerebrovascular function in a mouse model of AD [44].

## 5. Conclusions

Overall, our findings highlight that ApoE acts as a multi-functional mediator in AD-related pathological processes. In an isoform-specific manner, ApoE regulates neuroinflammation and neurotoxicity through direct actions on microglia, astrocytes, and neurons. In general, ApoE4 promotes pro-inflammatory and neurotoxic processes, while ApoE2 acts in a neuroprotective manner, highlighting the differential risk associated with these ApoE isoforms in the development of AD. Additionally, these findings suggest that targeting ApoE-related signaling may be potential therapeutic strategy for mitigating multiple AD-related pathological processes in individuals who carry the ApoE4 allele.

## Figures and Tables

**Table 1:** |SH-SY5Y viability and toxicity following treatment with ApoE2/3/4 in vitro. Human neuroblastoma cell line (SH-SY5Y) was grown in vitro and treated for 24 h with 100 μM cholesterol (control) with or without recombinant ApoE2/3/4 (20 nM). Following treatment, cellular viability was measured using MTS and cellular release of lactate dehydrogenase (LDH) was assessed as a measure of cellular toxicity. Data are represented as percent change versus control Mean ± SEM, N = 3, * *p* < 0.05 vs. Control, ** *p* < 0.01 vs. Control, ^^^ *p* < 0.001 vs. ApoE2, ^###^ *p* < 0.001 vs. ApoE3.

Treatment Group	ApoE2	ApoE3	ApoE4
Cellular Viability (MTS)	+14.9 ± 0.663 **	+9.7 ± 2.780	−9.7 ± 4.202 ^^^^,###^
Cellular Toxicity (LDH)	+2.6 ± 3.046	+2.7 ± 4.156	+14 ± 4.164 *

## Data Availability

The data used in this study are available from the corresponding author upon reasonable request.
